# Supplementary material for: Presynaptic LRP4 promotes synapse number and function of excitatory CNS neurons
Source: eLife. 2017 Jun 13;6:e27347. doi: 10.7554/eLife.27347 (PMC5469616; doi:10.7554/eLife.27347)
Supplement: Supplementary file 1. — Full genotype information for each image or analyzed condition. Notation (+; +; +; +) follows standard Drosophila genetics, corresponding to the X, 2nd, 3rd, and 4th chromosomes. Corresponding figure panels are listed as well for each genotype. DOI: http://dx.doi.org/10.7554/eLife.27347.021 [file elife-27347-supp1.docx]

**Supplementary File 1. Table of genotypes for all experimental conditions.**

Full genotype information for each image or analyzed condition. Notation (*+; +; +; +*) follows standard *Drosophila* genetics, corresponding to the X, 2^nd^, 3^rd^, and 4^th^ chromosomes. Corresponding figure panels are listed as well for each genotype.

| Figure | Panel | Genotype |
| --- | --- | --- |
| 1 | *B* | *+; +; +; +* |
|  | *C* | *lrp4^dalek^ / Y; +; +; +*. |
|  | *D* | w, UAS-Syt-HA / Y*; +; lrp4-GAL4 / +; +* |
|  | *E* | w, *pebbled-GAL4 / Y; +; UAS-LRP4-HA / +; +* |
|  | *F* |  |
|  | *G* | *w; UAS-mCD8-GFP / +; lrp4-GAL4 / +; +* |
|  | *H* |  |
|  | *I* |  |
|  | *J* |  |
|  | *K* |  |
| 1-2 | *B* | + / + = *+; +; +; +* |
|  |  | + / - = *lrp4^dalek^ / +; +; +; +* |
|  |  | - / - = *lrp4^dalek^; +; +; +* |
|  | *C* | *+; +; lrp4-GAL4 / UAS-LRP4-HA; +* |
|  | *D* | *eyFLP^3.5^ / Y; UAS-FRT-STOP-FRT-mCD8-GFP / +; lrp4-GAL4 / +; +* |
|  | *E* | *+ / Y; GH146-FLP, UAS-FRT-STOP-FRT-mCD8-GFP / +; lrp4-GAL4 / +; +* |
| 1-3 | *A* | *+; +; +; +* |
|  | *B* | *pebbled-GAL4 / Y; UAS-Brp-Short-mStraw / +; +; +* |
| 2 | *B* | *UAS-Dcr2 / Y; UAS-Brp-Short-mStraw, UAS-mCD8-GFP / +; Or47b-GAL4 / +; +* |
|  | *C* | *lrp4^dalek^ / Y; UAS-Brp-Short-mStraw, UAS-mCD8-GFP / UAS-Dcr2; Or47b-GAL4 / +; +* |
|  | *D* | *UAS-Dcr2 / Y; UAS-Brp-Short-mStraw / UAS-lrp4-RNAi 2; Or47b-GAL4 / +; +* |
|  | *E* | *+ / Y; UAS-Brp-Short-mStraw / +; UAS-LRP4-HA / Or47b-GAL4; +*. |
|  | *F* | *+ / Y; Mz19-GAL4 / +; UAS-Dα7-EGFP / +; +* |
|  | *G* | *lrp4^dalek^ / Y*; *Mz19-GAL4 / +; UAS-Dα7-EGFP / +; +* |
|  | *H* | *Control* = *UAS-Dcr2 / Y; UAS-Brp-Short-mStraw, UAS-mCD8-GFP / +; Or47b-GAL4 / +; +* |
|  |  | *lrp4^dalek^* = *lrp4^dalek^ / Y; UAS-Brp-Short-mStraw, UAS-mCD8-GFP / UAS-Dcr2; Or47b-GAL4 / +; +* |
|  |  | *lrp4^IR-1^* = *UAS-Dcr2 / Y; UAS-Brp-Short-mStraw / UAS-lrp4-RNAi 1; Or47b-GAL4 / +; +* and  *UAS-Dcr2 / Y; UAS-mCD8-GFP / UAS-lrp4-RNAi 1; Or47b-GAL4 / +; +* |
|  |  | *lrp4^IR-2^* = *UAS-Dcr2 / Y; UAS-Brp-Short-mStraw / UAS-lrp4-RNAi 2; Or47b-GAL4 / +; +* and  *UAS-Dcr2 / Y; UAS-mCD8-GFP / UAS-lrp4-RNAi 2; Or47b-GAL4 / +; +* |
|  |  | *lrp4^IR-3^* = *UAS-Dcr2 / Y; UAS-Brp-Short-mStraw / +; Or47b-GAL4 / UAS-lrp4-RNAi 3; +* and  *UAS-Dcr2 / Y; UAS-mCD8-GFP / +; Or47b-GAL4 / UAS-lrp4-RNAi 3; +* |
|  |  | *lrp4^IR-4^* = *UAS-Dcr2 / Y; UAS-Brp-Short-mStraw / +; Or47b-GAL4 / UAS-lrp4-RNAi 4; +* and  *UAS-Dcr2 / Y; UAS-mCD8-GFP / +; Or47b-GAL4 / UAS-lrp4-RNAi 4; +* |
|  |  | *LRP4^OE^* = *UAS-Dcr2 / Y; UAS-Brp-Short-mStraw / +; UAS-LRP4-HA / Or47b-GAL4; +* and  *UAS-Dcr2 / Y; UAS-mCD8-GFP / +; UAS-LRP4-HA / Or47b-GAL4; +*. |
|  | *I* | *Control* = *+ / Y; Mz19-GAL4, UAS-3xHA-mTDT / +; UAS-Dα7-EGFP / +; +* |
|  |  | *lrp4^dalek^* = *lrp4^dalek^ / Y; Mz19-GAL4, UAS-3xHA-mTDT / +; UAS-Dα7-EGFP / +; +* |
| 2-1 | *A* | *UAS-Dcr2 / Y; UAS-Brp-Short-mStraw, UAS-mCD8-GFP / +; Or47b-GAL4 / +; +* |
|  | *B* | *UAS-Dcr2 / Y; UAS-Brp-Short-mStraw / UAS-lrp4-RNAi 1; Or47b-GAL4 / +; +* |
|  | *C* | *UAS-Dcr2 / Y; UAS-Brp-Short-mStraw / +; Or47b-GAL4 / UAS-lrp4-RNAi 3; +* |
|  | *D* | *UAS-Dcr2 / Y; UAS-Brp-Short-mStraw / +; Or47b-GAL4 / UAS-lrp4-RNAi 4; +* |
|  | *E* | *UAS-Dcr2 / Y; UAS-Brp-Short-mStraw, UAS-mCD8-GFP / +; Or47b-GAL4 / +; +* |
|  | *F* | *lrp4^dalek^ / Y; UAS-Brp-Short-mStraw, UAS-mCD8-GFP / UAS-Dcr2; Or47b-GAL4 / +; +* |
|  | *G* | *UAS-Dcr2 / Y; UAS-mCD8-GFP / UAS-lrp4-RNAi 1; Or47b-GAL4 / +; +* |
|  | *H* | *UAS-Dcr2 / Y; UAS-mCD8-GFP / UAS-lrp4-RNAi 2; Or47b-GAL4 / +; +* |
|  | *I* | *UAS-Dcr2 / Y; UAS-mCD8-GFP / +; Or47b-GAL4 / UAS-lrp4-RNAi 3; +* |
|  | *J* | *UAS-Dcr2 / Y; UAS-mCD8-GFP / +; Or47b-GAL4 / UAS-lrp4-RNAi 4; +* |
|  | *K* | *UAS-Dcr2 / Y; UAS-mCD8-GFP / +; UAS-LRP4-HA / Or47b-GAL4; +* |
|  | *L* | *+ / Y; Mz19-GAL4, UAS-3xHA-mTDT / +; UAS-Dα7-EGFP / +; +* |
|  | *M* | *lrp4^dalek^ / Y; Mz19-GAL4, UAS-3xHA-mTDT / +; UAS-Dα7-EGFP / +; +* |
| 2-2 | *A* | *UAS-Dcr2 / +; UAS-Brp-Short-mStraw, UAS-mCD8-GFP / +; Or47b-GAL4 / +; +* |
|  | *B* | *UAS-Dcr2 / +; UAS-Brp-Short-mStraw / UAS-lrp4-RNAi 1; Or47b-GAL4 / +; +* |
|  | *C* | *UAS-Dcr2 / +; UAS-Brp-Short-mStraw / UAS-lrp4-RNAi 2; Or47b-GAL4 / +; +* |
|  | *D* | *UAS-Dcr2 / +; UAS-Brp-Short-mStraw / +; Or47b-GAL4 / UAS-lrp4-RNAi 3; +* |
|  | *E* | *UAS-Dcr2 / +; UAS-Brp-Short-mStraw / +; Or47b-GAL4 / UAS-lrp4-RNAi 4; +* |
|  | *F* | *UAS-Dcr2 / +; UAS-Brp-Short-mStraw, UAS-mCD8-GFP / +; Or47b-GAL4 / +; +* |
|  | *G* | *UAS-Dcr2 / +; UAS-mCD8-GFP / UAS-lrp4-RNAi 1; Or47b-GAL4 / +; +* |
|  | *H* | *UAS-Dcr2 / +; UAS-mCD8-GFP / UAS-lrp4-RNAi 2; Or47b-GAL4 / +; +* |
|  | *I* | *UAS-Dcr2 / +; UAS-mCD8-GFP / +; Or47b-GAL4 / UAS-lrp4-RNAi 3; +* |
|  | *J* | *UAS-Dcr2 / +; UAS-mCD8-GFP / +; Or47b-GAL4 / UAS-lrp4-RNAi 4; +* |
|  | *K* | *UAS-Dcr2 / +; UAS-Brp-Short-mStraw / +; UAS-LRP4-HA / Or47b-GAL4; +* |
|  | *L* | *UAS-Dcr2 / +; UAS-mCD8-GFP / +; UAS-LRP4-HA / Or47b-GAL4; +* |
|  | *M* | *Control* = *UAS-Dcr2 / Y; UAS-Brp-Short-mStraw, UAS-mCD8-GFP / +; Or47b-GAL4 / +; +* |
|  |  | *lrp4^dalek^* = *lrp4^dalek^ / Y; UAS-Brp-Short-mStraw, UAS-mCD8-GFP / UAS-Dcr2; Or47b-GAL4 / +; +* |
|  |  | *lrp4^IR-1^* = *UAS-Dcr2 / Y; UAS-Brp-Short-mStraw / UAS-lrp4-RNAi 1; Or47b-GAL4 / +; +* and  *UAS-Dcr2 / Y; UAS-mCD8-GFP / UAS-lrp4-RNAi 1; Or47b-GAL4 / +; +* |
|  |  | *lrp4^IR-2^* = *UAS-Dcr2 / Y; UAS-Brp-Short-mStraw / UAS-lrp4-RNAi 2; Or47b-GAL4 / +; +* and  *UAS-Dcr2 / Y; UAS-mCD8-GFP / UAS-lrp4-RNAi 2; Or47b-GAL4 / +; +* |
|  |  | *lrp4^IR-3^* = *UAS-Dcr2 / Y; UAS-Brp-Short-mStraw / +; Or47b-GAL4 / UAS-lrp4-RNAi 3; +* and  *UAS-Dcr2 / Y; UAS-mCD8-GFP / +; Or47b-GAL4 / UAS-lrp4-RNAi 3; +* |
|  |  | *lrp4^IR-4^* = *UAS-Dcr2 / Y; UAS-Brp-Short-mStraw / +; Or47b-GAL4 / UAS-lrp4-RNAi 4; +* and  *UAS-Dcr2 / Y; UAS-mCD8-GFP / +; Or47b-GAL4 / UAS-lrp4-RNAi 4; +* |
|  |  | *LRP4^OE^* = *UAS-Dcr2 / Y; UAS-Brp-Short-mStraw / +; UAS-LRP4-HA / Or47b-GAL4; +* and  *UAS-Dcr2 / Y; UAS-mCD8-GFP / +; UAS-LRP4-HA / Or47b-GAL4; +*. |
| 2-3 | *A* | *UAS-Dcr2 / Y; AM29-GAL4, UAS-Brp-Short-mStraw / UAS-mCD8-GFP; +; +* |
|  | *B* | *UAS-Dcr2 / Y; AM29-GAL4, UAS-Brp-Short-mStraw / UAS-lrp4-RNAi 1; +; +* |
|  | *C* | *UAS-Dcr2 / Y; AM29-GAL4, UAS-Brp-Short-mStraw / UAS-lrp4-RNAi 2; +; +* |
|  | *D* | *UAS-Dcr2 / Y; UAS-Brp-Short-mStraw, UAS-mCD8-GFP / +; Or88a-GAL4 / +; +* |
|  | *E* | *UAS-Dcr2 / Y; UAS-Brp-Short-mStraw / UAS-lrp4-RNAi 1; Or88a-GAL4 / +; +* |
|  | *F* | *UAS-Dcr2 / Y; UAS-Brp-Short-mStraw / UAS-lrp4-RNAi 2; Or88a-GAL4 / +; +* |
|  | *G* | *UAS-Dcr2 / Y; UAS-Brp-Short-mStraw, UAS-mCD8-GFP / +; Or67d-GAL4 / +; +* |
|  | *H* | *UAS-Dcr2 / Y; UAS-Brp-Short-mStraw / UAS-lrp4-RNAi 1; Or67d-GAL4 / +; +* |
|  | *I* | *UAS-Dcr2 / Y; UAS-Brp-Short-mStraw / UAS-lrp4-RNAi 2; Or67d-GAL4 / +; +* |
|  | *J* | DL4 and DM6 = same as S6a-c |
|  |  | VA1d = same as S6d-f |
|  |  | DA1 = same as S6g-i |
| 2-4 | *A* | *UAS-Dcr2 / Y; UAS-3xHA-mtdT / +; Or67d-GAL4 / UAS-DSyd1-GFP; +* |
|  | *B* | *UAS-Dcr2 / Y; UAS-lrp4-RNAi 1 / +; Or67d-GAL4 / UAS-DSyd1-GFP; +* |
|  | *C* | *UAS-Dcr2 / Y; UAS-lrp4-RNAi 2 / +; Or67d-GAL4 / UAS-DSyd1-GFP; +* |
|  | *D* | Control = *UAS-Dcr2 / Y; UAS-3xHA-mtdT / +; Or67d-GAL4 / UAS-DSyd1-GFP; +* |
|  |  | lrp4^IR-1^ = *UAS-Dcr2 / Y; UAS-lrp4-RNAi 1 / +; Or67d-GAL4 / UAS-DSyd1-GFP; +* |
|  |  | lrp4^IR-2^ = *UAS-Dcr2 / Y; UAS-lrp4-RNAi 2 / +; Or67d-GAL4 / UAS-DSyd1-GFP; +* |
| 3 |  | *Control* = *+ / Y; +; +; +*  *lrp4^dalek^* = *lrp4^dalek^ / Y; +; +; +* |
| 4 | *A* | UAS-Dcr2 / Y; GAD1-GAL4 / UAS-Brp-Short-mStraw, UAS-mCD8-GFP; +; + |
|  | *B* | *lrp4^dalek^ / Y; GAD1-GAL4 / UAS-Brp-Short-mStraw, UAS-mCD8-GFP; UAS-Dcr2 / +; +* |
|  | *C* | *UAS-Dcr2 / Y; GAD1-GAL4 / UAS-Brp-Short-mStraw; UAS-LRP4-HA / +; +* |
|  | *D* | *Control* = *UAS-Dcr2 / Y; GAD1-GAL4 / UAS-Brp-Short-mStraw, UAS-mCD8-GFP; +; +* |
|  |  | *lrp4^dalek^* = *lrp4^dalek^ / Y; GAD1-GAL4 / UAS-Brp-Short-mStraw, UAS-mCD8-GFP; UAS-Dcr2 / +; +* |
|  |  | *lrp4^IR-1^* = *UAS-Dcr2 / Y; GAD1-GAL4, UAS-Brp-Short-mStraw / UAS-lrp4-RNAi 1; +; +* and  *UAS-Dcr2 / Y; GAD1-GAL4 / UAS-lrp4-RNAi 1; UAS-mCD8-GFP / +; +* |
|  |  | *lrp4^IR-2^* = *UAS-Dcr2 / Y; GAD1-GAL4, UAS-Brp-Short-mStraw / UAS-lrp4-RNAi 2; +; +* and  *UAS-Dcr2 / Y; GAD1-GAL4 / UAS-lrp4-RNAi 2; UAS-mCD8-GFP / +; +* |
|  |  | *LRP4^OE^* = *UAS-Dcr2 / Y; UAS-Brp-Short-mStraw / GAD1-GAL4; UAS-LRP4-HA / +; +* and  *UAS-Dcr2 / Y; UAS-mCD8-GFP / GAD1-GAL4; UAS-LRP4-HA / +; +* |
| 5 | *B* | *+ / Y; Mz19-GAL4 / UAS-mCD8-GFP, UAS-Brp-Short-mStraw; UAS-Dcr2 / +; +* |
|  | *C* | *lrp4^dalek^ / Y; Mz19-GAL4 / UAS-mCD8-GFP, UAS-Brp-Short-mStraw; UAS-Dcr2 / +; +* |
|  | *D* | *UAS-Dcr2 / Y; UAS-Brp-Short-mStraw, UAS-mCD8-GFP / +; Mz699-GAL4 / +; +* |
|  | *E* | *lrp4^dalek^ / Y; UAS-Brp-Short-mStraw, UAS-mCD8-GFP / UAS-Dcr2; Mz699-GAL4 / +; +* |
|  | *F* | Control = *+ / Y; Mz19-GAL4 / UAS-mCD8-GFP, UAS-Brp-Short-mStraw; UAS-Dcr2 / +; +* |
|  |  | *lrp4^dalek^* = *lrp4^dalek^ / Y; Mz19-GAL4 / UAS-mCD8-GFP, UAS-Brp-Short-mStraw; UAS-Dcr2 / +; +* |
|  |  | *lrp4^IR-1^* = *+ / Y; Mz19-GAL4, UAS-mCD8-GFP / UAS-lrp4-RNAi 1; UAS-Brp-Short-mStraw / +; +* |
|  |  | *lrp4^IR-2^* = *+ / Y; Mz19-GAL4, UAS-mCD8-GFP / UAS-lrp4-RNAi 2; UAS-Brp-Short-mStraw / +; +* |
|  | *G* | Control = *+ / Y; UAS-Brp-Short-mStraw, UAS-mCD8-GFP / +; Mz699-GAL4 / UAS-Dcr2; +; +* |
|  |  | *lrp4^dalek^* = *lrp4^dalek^ / Y; UAS-Brp-Short-mStraw, UAS-mCD8-GFP / +; Mz699-GAL4 / UAS-Dcr2; +* |
|  |  | *lrp4^IR-1^* = *+ / Y; UAS-mCD8-GFP, UAS-Brp-Short-mStraw / UAS-lrp4-RNAi 1; Mz699-GAL4 / +; +* |
|  |  | *lrp4^IR-2^* = *+ / Y; UAS-mCD8-GFP, UAS-Brp-Short-mStraw / UAS-lrp4-RNAi 2; Mz699-GAL4 / +; +* |
|  |  | *LRP4^OE^* = *+ / Y; UAS-mCD8-GFP, UAS-Brp-Short-mStraw / +; Mz699-GAL4 / UAS-LRP4-HA; +* |
| 5-1 | *A* | *+ / Y; Mz19-GAL4 / UAS-mCD8-GFP, UAS-Brp-Short-mStraw; UAS-Dcr2 / +; +* |
|  | *B* | *+ / Y; Mz19-GAL4, UAS-mCD8-GFP / UAS-lrp4-RNAi 1; UAS-Brp-Short-mStraw / +; +* |
|  | *C* | *+ / Y; Mz19-GAL4, UAS-mCD8-GFP / UAS-lrp4-RNAi 2; UAS-Brp-Short-mStraw / +; +* |
|  | *D* | *+ / Y; UAS-Brp-Short-mStraw, UAS-mCD8-GFP / +; Mz699-GAL4 / UAS-Dcr2; +; +* |
|  | *E* | *+ / Y; UAS-mCD8-GFP, UAS-Brp-Short-mStraw / UAS-lrp4-RNAi 1; Mz699-GAL4 / +; +* |
|  | *F* | *+ / Y; UAS-mCD8-GFP, UAS-Brp-Short-mStraw / UAS-lrp4-RNAi 2; Mz699-GAL4 / +; +* |
|  | *G* | *+ / Y; UAS-mCD8-GFP, UAS-Brp-Short-mStraw / +; Mz699-GAL4 / UAS-LRP4-HA; +* |
| 6 | *B* | Column 1 *= pebbled-GAL4 / Y; UAS-mCD8-GFP / +; UAS-mCD8-GFP / +; +* |
|  |  | Column 2 *= + / Y; UAS-mCD8-GFP / UAS-lrp4-RNAi 1; UAS-mCD8-GFP / +; +* |
|  |  | Column 3 = *+ / Y; UAS-mCD8-GFP / UAS-lrp4-RNAi 2; UAS-mCD8-GFP / +; +* |
|  |  | Column 4 = *+ / Y; UAS-mCD8-GFP / +; UAS-mCD8-GFP / UAS-lrp4-RNAi 3; +* |
|  |  | Column 5 = *+ / Y; UAS-mCD8-GFP / +; UAS-mCD8-GFP / UAS-lrp4-RNAi 4; +* |
|  |  | Column 6 = *pebbled-GAL4 / Y; UAS-lrp4-RNAi 1 / +; UAS-mCD8-GFP / +; +* |
|  |  | Column 7 = *pebbled-GAL4 / Y; UAS-lrp4-RNAi 2 / +; UAS-mCD8-GFP / +; +* |
|  |  | Column 8 = *pebbled-GAL4 / Y; UAS-mCD8-GFP / +; UAS-lrp4-RNAi 3 / +; +* |
|  |  | Column 9 = *pebbled-GAL4 / Y; UAS-mCD8-GFP / +; UAS-lrp4-RNAi 4/ +; +* |
|  |  | Column 10 = *+ / Y; UAS-mCD8-GFP / UAS-GABA_B_R2-RNAi; UAS-mCD8-GFP / +; +* |
|  |  | Column 11 = *pebbled-GAL4 / Y; UAS-mCD8-GFP / UAS-GABA_B_R2-RNAi; UAS-mCD8-GFP / +; +* |
|  |  | Column 12 = *+ / Y; UAS-GABA_B_R2-RNAi / UAS-lrp4-RNAi 2; UAS-mCD8-GFP / +; +* |
|  |  | Column 13 = *pebbled-GAL4 / Y; UAS-lrp4-RNAi 2 / UAS-GABA_B_R2-RNAi; +; +* |
| 7 | *A* | *+ / Y; +; +; +* |
|  | *B* | *lrp4^dalek^ / Y; +; +; +* |
|  | *C* | *+ / Y; +; srpk^atc^; +* |
|  | *D* | *+ / Y; UAS-Brp-Short-mStraw / UAS-venus-SRPK79D-28; Or47b-GAL4 / +; +* |
|  | *E* | *pebbled-GAL4 / Y; UAS-venus-SRPK79D-28 / +; LRP4-HA / +; +* |
|  | *F* | *+ / Y; Or47b-GAL4 / UAS-venus-SRPK79D-28; +; +* |
|  | *G* | *lrp4^dalek^ / Y; Or47b-GAL4 / UAS-venus-SRPK79D-28; +; +* |
|  | *H* | *Control* = *+ / Y; Or47b-GAL4 / UAS-venus-SRPK79D-28; +; +* |
|  |  | *lrp4^dalek^* = *lrp4^dalek^ / Y; Or47b-GAL4 / UAS-venus-SRPK79D-28; +; +* |
|  | *I* | *pebbled-GAL4 / Y; UAS-venus-SRPK79D-28 / +; LRP4-HA / +; +* |
|  | *J* |  |
| 7-1 | *A* | *pebbled-GAL4 / Y; UAS-SRPK79D-28 / UAS-Dcr2; +; +* |
|  | *B* | *pebbled-GAL4 / Y; UAS-Dcr2 / +; UAS-LRP4-HA/ +; +* |
|  | *C* | *pebbled-GAL4 / Y; UAS-SRPK79D-28 / +; UAS-LRP4-HA / +; +* |
|  | *D* |  |
| 8 | *A* | *UAS-Dcr2 / Y; UAS-Brp-Short-mStraw, UAS-mCD8-GFP / +; Or47b-GAL4 / +; +* |
|  | *B* | *UAS-Dcr2 / Y; UAS-Brp-Short-mStraw / +; Or47b-GAL4 / UAS-srpk79D-RNAi; +* |
|  | *C* | *lrp4^dalek^ / Y; UAS-Brp-Short-mStraw, UAS-mCD8-GFP / UAS-Dcr2; Or47b-GAL4 / +; +* |
|  | *D* | *lrp4^dalek^ / Y; UAS-Brp-Short-mStraw, UAS-mCD8-GFP / +; Or47b-GAL4 / UAS-venus-SRPK79D-1A; +* |
|  | *E* | *Control* = *UAS-Dcr2 / Y; UAS-Brp-Short-mStraw, UAS-mCD8-GFP / +; Or47b-GAL4 / +; +* |
|  |  | *srpk^RNAi^* = *UAS-Dcr2 / Y; UAS-Brp-Short-mStraw / +; Or47b-GAL4 / UAS-srpk79D-RNAi; +* |
|  |  | srpk^RNAi^ + LRP4^OE^ = *UAS-Dcr2 / Y; Or47b-GAL4 / UAS-srpk79D-RNAi; UAS-LRP4-HA / +; +* |
|  |  | *lrp4^dalek^* = *lrp4^dalek^ / Y; UAS-Brp-Short-mStraw, UAS-mCD8-GFP / UAS-Dcr2; Or47b-GAL4 / +; +* |
|  |  | *SRPK^OE^* = *+ / Y; UAS-Brp-Short-mStraw, UAS-mCD8-GFP / +; Or47b-GAL4 / UAS-venus-SRPK79D-1A; +* |
|  |  | *lrp4^dalek^ + SRPK^OE^ = lrp4^dalek^ / Y; UAS-Brp-Short-mStraw, UAS-mCD8-GFP / +; Or47b-GAL4 / UAS-venus-SRPK79D-1A; +* |
|  | *F* | Column 1 = *pebbled-GAL4 / Y; UAS-mCD8-GFP / +; UAS-mCD8-GFP / +; +* |
|  |  | Column 2 = *+ / Y; UAS-srpk79D-RNAi / +; UAS-mCD8-GFP / +; +* |
|  |  | Column 3 = *+ / Y; UAS-mCD8-GFP / UAS-lrp4-RNAi 1; UAS-mCD8-GFP / +; +* |
|  |  | Column 4 = *+ / Y; UAS-mCD8-GFP / +; UAS-mCD8-GFP / UAS-venus-SRPK79D-1A; +* |
|  |  | Column 5 = *+ / Y; UAS-lrp4-RNAi 1 / +; UAS-mCD8-GFP / UAS-venus-SRPK79D-1A; +* |
|  |  | Column 6 = *pebbled-GAL4 / Y; UAS-mCD8-GFP / +; UAS-venus-SRPK79D-1A / +; +* |
|  |  | Column 7 = *pebbled-GAL4 / Y; UAS-srpk79D-RNAi / +; UAS-mCD8-GFP / +; +* |
|  |  | Column 8 = *pebbled-GAL4 / Y; UAS-lrp4-RNAi 1 / +; UAS-mCD8-GFP / +; +* |
|  |  | Column 9 = *pebbled-GAL4 / Y; UAS-lrp4-RNAi 1 / +; UAS-venus-SRPK79D-1A / +; +* |
| 8-1 | *A* | *+; +; +; +* |
|  | *B* | *lrp4^dalek^ / +; +; +; +* |
|  | *C* | *+; +; srpk^atc^ / +; +* |
|  | *D* | *lrp4^dalek^ / +; +; srpk^atc^ / +; +* |
|  | *E* | *+ / + = +; +; +; +* |
|  |  | *lrp4 + / - srpk + / + = lrp4^dalek^ / +; +; +; +* |
|  |  | *lrp4 + / + srpk + / - = +; +; srpk^atc^ / +; +* |
|  |  | *lrp4 + / - srpk + / - = lrp4^dalek^ / +; +; srpk^atc^ / +; +* |
